# Supplementary material for: Are carbon emissions trading and green financial instruments synergistic? -Comprehensive quantitative research based on content analysis
Source: PLoS One. 2024 Mar 7;19(3):e0298601. doi: 10.1371/journal.pone.0298601 (PMC10919601; doi:10.1371/journal.pone.0298601)
Supplement: S1 Data — (ZIP) [file pone.0298601.s001.zip › Data/地级市Z_F工作报告.zip!/║┌┴·╜¡/╥┴┤║╩╨/└┤╫╘╣1⁄2╓┌║┼í╛┬φ┐╦╩2╛▌═°í┐_2011.docx]

各位代表：

现在，我代表市人民政府作工作报告，请予审议，并请市政协委员和列席会议的同志提出意见。

一、去年及“十一五”工作回顾

过去的一年，是我市发展进程中极不平凡的一年。在市委的领导下，全市人民以科学发展观为指导，围绕加快解放思想、学习赶超先进、努力实现伊春科学发展新跨越的总体要求，振奋精神，抢抓机遇，真抓实干，圆满完成了市十二届五次人代会确定的目标任务，伊春的经济社会及生态建设取得了重大进展。

（一）经济运行质量稳步提高。全市生产总值完成202.4亿元，跨上200亿元台阶,同比增长15.7%。财政收入实现20.3亿元,突破20亿元大关，同比增长38.3%，为预算的171.2%，其中地方财政收入实现12.8亿元，同比增长45.9%,为预算的198.3%。规模以上工业实现增加值53.6亿元，同比增长34.5%。企业经济效益综合指数达到153.2，同比提高7.6个百分点。农业生产战胜了春季低温多雨的灾害天气夺取丰收，粮食总产量达到80.3万吨，同比增长16%。五大优势特色产业实现增加值86.3亿元，同比增长27.6%。森林生态旅游业得到提升，建成和完善了乌马河西岭狩猎场、室内冰壶运动场等一批冬季旅游设施项目。全年接待游客419万人次，实现旅游收入25.2亿元，同比分别增长9.9%和31.6%。森林食品业势头良好，实现增加值14.8亿元，同比增长11.5%。金融、保险、信息、物流业得到了新发展。消费市场稳中趋旺，住房、汽车、旅游等消费不断扩大，社会消费品零售总额实现56.7亿元，同比增长18.8%。城镇居民人均可支配收入达到10317元，农民人均纯收入达到7280元，同比分别增长9.1%、18.6%。“十一五”节能减排指标全面完成。万元GDP综合能耗同比下降7.6%，二氧化硫排放量削减率达7.1%，化学耗氧量下降6.1%。经济运行的稳定性、协调性、科学性明显增强。

（二）生态建设深入推进。认真落实严管林措施，开展了治理林地“三乱”行动，收回林地643公顷。延长了林蛙停捕期，加强了对珍稀树种和野生鸟类、鱼类、蓝莓等动植物的保护，红松保护基金已募集3030万元。建立了木材网上统一销售制度，有效遏制了超采行为。积极开展“大造林”行动和森林抚育补贴试点，造林绿化34.6万亩，抚育试点40.6万亩，计划任务全面完成。首次开展了伊春林区森林与湿地资源价值评估，为今后考量生态建设成果和实施碳汇交易奠定了基础。

（三）发展基础显著增强。固定资产投资完成153.6亿元，增幅达到50.2%，在全省名列前茅。项目建设取得重大进展。落实市级重点项目60个，投资额相当于前三年之和。其中续建和新开工项目49个，亿元以上项目38个，重大产业项目42个。西钢技改、浩水扩能等重点项目相继投产，五星级酒店、液化工业气体、铅冶炼、蓝莓产业化等项目进展顺利，鹿鸣钼矿开发前期工作已经展开。城市基础设施趋于完善。强力推进了伊绥高速、前嫩高速伊春段建设，黑嘉、绥嘉沿边公路主体工程建成通车，改造农村公路288.5公里。深入实施“三供两治”工程，投资5亿多元完成了供热、供水和污水处理基础设施建设，中心城新建小区全部规划了供气系统，城市污水、垃圾处理厂（场）投入运行。投资4.7亿元完成了新青输变电等电网建设工程。全市绿地率达到50.23%，人均公共绿地面积新增1.79平方米，达到27.2平方米。小城镇和旅游名镇建设全面推进。将旅游开发与棚户区改造、生态移民、烧柴革命相结合，完成了美溪松岭等13个林场所1885户居民的搬迁转移，乌马河“西岭模式”得到了中央领导和省委省政府的充分肯定。

（四）改革开放不断深化。国有林权制度改革试点扎实推进，综合效应继续显现。国企改革迈出新步伐，光明集团重组工作稳步实施，与32户企业联名联牌发展，龙头带动力明显增强；浩水公司成功并购重组晨明水泥厂和美溪建材公司。政府机构改革顺利完成，医药卫生、文化体制改革有序推进。金融体制改革取得新突破，农村信用社改革开始启动，龙江银行、海通证券的分支机构在我市挂牌运营。以招商引资、对俄经贸合作为重点，对内对外开放迈出新步伐。华能、华仪、中铁、永同昌集团等一批战略投资者落户伊春。全年引进国内资金132.6亿元，同比增长134%；实际利用外资4540万美元，同比增长58%。海关检验检疫机构正式进驻中心区，外贸企业进出口更加方便，全年外贸进出口完成3.02亿美元，同比增长36.3%。乌马河、上甘岭对俄采伐合作和嘉荫对俄农业开发顺利推进。成功举办了森林产品博览会和第十届森林旅游节等活动，我市的对外知名度、影响力进一步扩大。

（五）社会事业全面进步。科技创新与应用水平进一步提高，取得市级以上科技成果18项，转化率达72.3%。教育事业均衡发展，学校布局调整循序推进，办学条件明显改善，伊春职业学院教学主楼等工程按时竣工，市职教中心等4所学校被评为国家级重点中等职业学校。医疗卫生基础设施建设全面加强，公共卫生服务体系日趋完善，社区卫生服务机构覆盖率达到90%。文化事业繁荣发展，公共文化设施覆盖面进一步扩大，文化创新能力得到提高，“大森林之声”等品牌文化活动影响力进一步增强。成功举办了首届中国伊春森林音乐会。体育事业蓬勃发展，竞技体育水平继续提高，群众体育活动深入开展，获得了2014年省十三运会承办权，举办了首届冰壶挑战赛。“四城联创”深入推进，创建国家卫生城市顺利通过技术评估。国防动员、民兵预备役、双拥和食品药品安全、质量监督工作进一步加强，妇女、儿童、残疾人权益得到有效保障，人口与计划生育、国土资源、民族宗教、外事侨务、史志档案等工作取得了新进展。第六次人口普查顺利完成。

（六）民生工程有效实施。2010年是我市民生投入最大、建设项目最多、惠及民众最广的一年。经多方筹资，全市用于民生的投入达60亿元，其中，地方财政投入47.6亿元，占总投入的79.4%，占财政总支出的59.3%。围绕年初确定的8项利民工程，市政府着力加大了督办检查和跟踪问效力度，承诺的32件实事中，棚户区及农村泥草房改造、城乡失业人员就业创业培训、小额担保贷款发放、城镇居民医保参保率、教育教学设备更新、普通高中贫困生和大学生资助、万名妇女健康普查等8件超额完成；新建和改扩建学校、医院、图书馆、标准化社区服务中心及提高各项保障标准、增加林业教师工资、提升物业管理水平等22件按计划完成或如期启动。由于发生了“8•16”特大爆炸事故，安全生产没有完成预期目标；增开航线虽获批准，但“8•24”空难发生后，国家提高了支线机场标准，需盲降系统建成后才能开通。市政府将继续努力，争取今年增开航线。随着这些民生工程的落实，全市公共服务进一步完善，人民群众的生活条件得到了新改善。

两起事故发生后，我们迅速组织抢险救援，全力医治受伤人员，妥善安置受灾群众，精心做好善后工作，将事故损失和影响降到了最低限度，赢得了人民群众的理解和支持，也得到了省委、省政府的充分肯定。

（七）民主法治建设持续加强。自觉接受人大、政协和人民群众的监督，全年办理人大常委会及代表提出的意见、建议157件，办理政协委员提案75件，办结率达到100%。积极推进依法治市，圆满完成了“五五”普法任务。不断深化“平安伊春”建设，继续保持了社会和谐稳定。大力转变政府职能，深入开展了“六型”机关创建和“百企万民评服务”等活动，全面深化了政务公开、村务公开、厂务公开和涉及民生重大事项的听证公示制度，认真落实《廉政准则》，着力加强了行政监察和审计监督工作，依法决策、民主决策、科学决策的水平进一步提高。

各位代表，去年各项工作任务的顺利完成，标志着我市“十一五”规划圆满收官。这五年，是我市对上争取硕果累累的五年。我们先后争取到了国有林权制度改革试点、全国首批资源枯竭城市、林区棚户区改造、生态功能区建设等一批事关伊春长远发展的重大政策，得到了国家政策性、财力性支持。这些政策的巨大效应会在“十二五”期间集中显现，将为我市的加快发展提供有力保障。这五年，是我市森林资源显著恢复的五年。我们积极创新理念，坚持保护与培育并举，严厉打击超采与盗伐，显著提升了森林防火能力，保障了森林资源安全，扭转了森林资源“消大于长”的逆向演替，历史性地实现了森林蓄积年净增500万立方米以上的良性循环。森林覆盖率由80.6%提高到83.4%，森林蓄积量由2.28亿立方米增加到2.5亿立方米。这五年，是我市经济转型加速推进的五年。森林生态旅游、绿色能源等五大优势特色产业增加值年均增长21%，占GDP的比重达到42.6%，五年提高了15个百分点。一些区局基本摆脱了对木材采伐的依赖。这五年，是我市基础设施建设取得重大突破的五年。林都机场通航运营，伊绥高速即将建成，兴安湖水库下闸蓄水，城乡道路交通条件显著改善，城市防洪工程、“三供两治”等一批事关长远发展、承载几代伊春人期盼与梦想的重大基础设施相继竣工或正在加紧建设，城乡面貌发生了历史性变化。这五年，是我市人民生活大幅改善的五年。我们大力实施了棚户区、泥草房改造和廉租房建设，改造棚户区503万平方米、泥草房6465户，建设廉租住房18275套，使10万多户居民改善了住房条件。累计建设农村饮水工程223处，解决了11.41万农村群众的饮水安全问题。先后解决了老知青、大集体、“五七工”的养老问题，补发了历史拖欠的林业职工工资和养老金，兑现了林区教师、公检法干警的国家机关和事业单位工资标准，增加了林业职工工资。城镇居民人均可支配收入、农民人均纯收入分别增加3266元和3489元。全面启动了城镇居民医疗保险，参保率达到92%。新农村建设成效显著，圆满完成了第二批试点任务。各项社会事业协调发展，社会保障和公共服务体系进一步完善。这五年，是我市知名度、影响力明显提升的五年。我们先后举办承办了保护红松国际研讨会、东北亚生态论坛、中国环境与健康发展论坛等重大会展活动，相继荣获了中国最具开发价值的旅游资源城市、全国社会治安综合治理优秀城市、全国未成年人思想道德建设先进城市、世界十佳和谐城市、各国驻华使馆最值得向世界推荐的中国生态旅游城市、全国绿化模范城市、2010年度中国十佳绿色城市等殊荣。这些成就，为我市“十二五”时期加快发展奠定了坚实基础。

五年的发展令人振奋，积累的经验弥足珍贵。工作中我们深切体会到：必须突出抓好森林资源的培育保护，严管资源，坚持“生态立市”不动摇；必须紧紧扭住经济建设这个中心，坚持“在保护中加快发展，在发展中兴市富民”不动摇；必须用改革创新的思路与方法解决我们面临的问题，坚持深化改革、扩大开放不动摇；必须将上级精神与我市实际紧密结合，坚持把争取政策支持与自力更生、真抓实干相统一不动摇；必须始终把实现好、维护好、发展好广大人民群众根本利益作为出发点和落脚点，坚持以人为本促进生态、经济、社会、文化全面协调可持续发展不动摇；必须适应工作任务的转变，不断加强政府自身建设，坚持科学行政、民主行政、依法行政和廉洁从政不动摇。这些经验，是我们今后推进伊春加快发展的宝贵精神财富。

各位代表，过去工作成绩的取得，是省委、省政府和市委正确领导的结果，是市人大、市政协大力支持的结果，是广大干部群众共同奋斗的结果。在此，我代表市人民政府，向全市广大工人、农民、知识分子、干部、离退休老同志、驻伊部队指战员，向各民主党派、人民团体和各界人士，致以崇高的敬意！向关心、支持伊春发展的中省直部门，向在我市创业的境内外投资者、建设者，表示衷心的感谢！

在肯定成绩的同时，必须清醒地认识到，我们的工作与人民群众的期望还有不少差距，工作中仍然存在一些问题需要解决。主要是：经济规模小，产业发展慢，群众收入低，部分群众生活困难；安全生产责任没有完全落实到位，发生了“8•16”特大事故，给人民群众生命财产造成了严重损失；政府职能转变还不到位，一些工作落实不够，办事效率不高，形式主义、作表面文章的现象仍然存在;资源依赖、政策依赖程度高，创新创业内生动力不足，发展环境仍需改善，等等。我们一定以高度负责的精神，重视解决存在的这些问题，扎扎实实地做好工作，不辜负人民的期望和重托。

二、关于全市第十二个五年规划纲要（草案）

根据市委十届十一次全会《建议》精神，市政府制定了《伊春市国民经济社会发展及生态建设第十二个五年规划纲要（草案）》（以下简称《纲要》），已提请大会审查。《纲要》分10个章节对全市未来五年发展建设的现实基础、目标任务和保证措施等进行了详尽的阐述和部署。下面，我重点报告两个问题。

（一）“十二五”时期经济社会发展及生态建设的基本原则和主要目标

结合当前我市的发展基础、势头以及国家和省的政策走向，《纲要》根据市委《建议》精神确定了基本原则，这就是：突出生态保护，加强和完善生态体系建设；大力调整产业结构，加速经济转型步伐；继续深化改革，不断创新体制机制；进一步扩大开放和招商引资，增强经济发展活力；推进科教振兴，实施人才强市战略；坚持以人为本，努力改善民生。同时，《纲要》提出了“十二五”时期发展的主要目标。这些目标既与全面建设小康社会的目标相衔接，又反映了经济社会发展及生态建设的阶段性特征和客观要求。

一是关于增长速度问题。《纲要》提出，到2015年，全市地区生产总值实现410亿元，年均增长15%，比2010年翻一番；地方财政收入达到26亿元，年均增长15%；固定资产投资达到690亿元，年均增长35%；农民人均纯收入达到12830元，年均增长12%；城镇居民人均可支配收入达到18182元，年均增长12%。这些目标是综合考虑我市的经济发展基础、潜力和面临的有利条件等各方面因素，根据需要和可能提出的。GDP增速确定为15%，既是经济增长目标，也是就业目标。我市是欠发达地区，目前在全省经济发展中的位次又非常靠后，所以，发展问题仍然是我们现阶段的主要矛盾，只有确定较高的发展速度，才能逐步实现赶超跨越，才能解决就业特别是新增就业等一系列现实问题。为此，在《纲要》之外，我市还内定了三年翻番的争取目标。固定资产投资年均增长35%，主要是着眼于产业项目、重大基础设施项目和民生项目建设，没有这样的增速，很难支撑经济加快发展。城乡居民收入“两个12%”的指标，主要是为了实现发展与改善民生同步，适应保障群众基本生活需要考虑的。这些目标现在看虽然稍高了一点，快了一点，但从近几年我市发展速度不断加快的趋势判断，如果没有特殊情况，经过努力是可以实现的。

二是关于生态建设问题。生态是伊春的“根”和“魂”。《纲要》的题目中加上“生态建设”的内容，提出了“十二五”期间森林覆被率稳定在83.4%，万元GDP综合能耗下降15%等目标，一方面体现了伊春的特色，彰显了我们高擎生态保护旗帜，实现科学发展、绿色崛起的信心和决心；另一方面，也体现了建设资源节约、环境友好型社会的要求，是扩大“绿色伊春”优势，追求现实和长远利益的需要。

(二)“十二五”时期的战略重点和主要任务

“十二五”时期，我市加快发展、加速转型的任务很艰巨。《纲要》在把握重点与一般、继承与创新、外因与内因关系的基础上，对“十二五”时期的生态建设、经济社会发展、改革开放等方面，都作出了全面部署，提出了明确的任务和措施。

一是加强生态保护。《纲要》把生态保护作为优先的战略任务。强调要积极落实大小兴安岭林区生态保护与经济转型规划，认真实施天保二期工程，巩固扩大生态保护成果，加强生态环境综合治理，积极发展低碳经济。全市上下必须一以贯之保生态，坚持不懈严管林，建立良性发展的森林生态体系，努力为国家乃至全球的生态建设不断作出新贡献。

二是加快产业发展。《纲要》提出，要以建立生态主导的林区经济体系为目标，继续发展五大接续产业，推进“4111工程”。充分发挥资源、环境优势和潜力，加快技术改造步伐，优化发展传统产业，稳步发展基础产业，大力发展特色产业，培育发展战略性新兴产业，做强转型支柱产业，推进产业结构调整和经济转型。这是针对我市产业规模小、总体素质不高、竞争力不强的现状而提出的一项重大任务。《纲要》对发展清洁工业、特色农业、低碳服务业等方面的主要任务和建设布局作了部署，规划了一批重大建设项目。对坚持科教兴市和人才强市战略，提高科技创新能力，发展高新技术产业，发挥科技、教育和人才的突出作用也作了安排。

三是深化改革开放。完成“十二五”期间的发展任务，必须坚定不移地全面深化改革和扩大开放。《纲要》提出，要坚持用改革的方法破解难题，推进重点领域和关键环节的改革，加快形成更具活力、更富效力、更加开放的体制机制，奠定科学发展、加快发展的体制基础。要深入推进国有林权制度改革、国有企业改革、各项财税改革和投资、行政及社会管理体制改革，优化非公有制经济发展环境。要面向国内外市场需求，实施“南联北开”、互利共赢的开放战略，开创全市外向型经济发展新局面。

四是构建和谐社会。《纲要》重点提出了改善人民生活，加快公共服务体系建设等方面的任务。强调要开展全民创业活动，促进充分就业，增加居民收入；完善提升社保体系和社会救助体系；扩大城乡居民消费，改善人居条件。要全面做好人口、教育、卫生、文化、体育等项工作。要推进“平安伊春”建设，加强社会主义政治文明和精神文明建设，确保在“十二五”早期进入全国文明城市行列。

各位代表，“十二五”规划纲要绘就了未来五年伊春的发展蓝图，表达了全市人民追求幸福，过上好日子的愿望。我们有充分的理由相信，五年后的伊春，一定会生态更加优良，产业更加兴旺，社会更加和谐，人民更加幸福，祖国林都必将以更快的步伐走向复兴，再创辉煌！

三、今年的主要工作

今年是“十二五”规划的开局之年，加快发展不仅对实现“十二五”目标意义深远，也是主动应对停止主伐给我市经济发展和群众收入带来影响的现实需要。

根据市委十届十一、十二次全会精神，今年政府工作的总体要求是：以科学发展观为统领，以保护生态为前提，以加快发展为核心，以转变经济发展方式为主线，紧紧抓住大小兴安岭林区生态保护与经济转型规划及天保二期工程实施等重大机遇，大上产业项目，大抓招商引资，着力改善民生，深化改革,扩大开放，巩固扩大生态保护成果，加快现代化园林城市建设，推动全市生态经济社会更好更快发展。

综合考虑各种因素，2011年的主要预期目标是：地区生产总值增长12%；全社会固定资产投资增长30%以上；规模以上工业增加值增长15%；地方财政收入增长15.2%；外贸进出口总额增长17%；城镇居民人均可支配收入和农民人均纯收入分别增长8%和10%；城镇登记失业率控制在4.5%以内；万元GDP综合能耗下降3%。为了实现上述目标，我们要重点做好以下五方面工作。

（一）培壮优势特色产业，在转变经济发展方式上实现新突破

今年是全省产业项目建设三年决战的首战之年，我们必须全力抓好重大产业项目建设，严格落实项目建设责任，进一步加快接续产业发展。全力推进投资119.5亿元的51个市级重点产业项目加快建设，促进产业项目向链条化、规模化、品牌化、高端化方向发展。做大做强优势工业。通过资源整合、要素重组，大力发展冶金建材、绿色能源、木材精深加工、制药等优势产业，推进工业结构优化升级。抓好西钢1260高炉等扩能项目建设，争取尽快竣工投产，实现年底新增产能100万吨。推进浩水日产4000吨水泥熟料新型干法生产线项目建设，达到年产300万吨以上的生产能力。突出发展矿产资源精深加工，全力抓好铁力鹿鸣钼矿开发项目、西林8万吨铅冶炼项目，必保达到既定进度，争取早日投产。大力推进与省地矿局在地质勘查与矿产开发方面的战略合作，加快矿产勘探进度。加快新青老白山、美溪圣旨山等风电场、汤旺河干流水电梯级开发及兴安新能源太阳能发电项目进程，努力实现风电场建设和入网同步进行。抓好葵花药业扩产改造等项目建设，力争尽快建成投产。继续做好板业、家具、小木制品的整合重组，重点以光明集团为龙头，促进企业之间联合与协作，做强林产工业。科学合理配置林木资源，切实保障木材精深加工企业原料供应。加快原料林基地建设，推进“林板一体化”进程。积极促进产业集聚发展，加快推进西林冶金循环经济园区、光明集团创业园区、南岔钼产业园区、嘉荫对俄加工园区建设，力争将中心区生态产业园区、铁力市农产品加工园区建设成为省级园区。加快发展农业和农村经济。大力发展优质粮食生产，加强“菜篮子”工程建设，利用西钢余热进行冬季蔬菜生产试点，不断扩大蔬菜、经济作物和森林食品、药材的种植养殖规模。加强森林食品基地建设，突出抓好蓝莓产业化发展，全年完成蓝莓种植3万亩。加快野生蓝莓的培育，提高产量。加快发展生态畜牧业，提高规模化、标准化水平。大力推进北药的产业化发展、食用菌产业素质升级和农业产业化经营，整合品牌资源，开展中国驰名商标和省著名商标争创活动，提高产品的附加值。强化龙头企业对基地的带动作用，鼓励农民创办各类经济合作组织。大力发展现代农业，提高装备水平，搞好抗旱水源和人畜饮水工程建设。深化铁力区域“1+5”合作模式和嘉荫场县共建模式，不断提高区域经济社会发展水平。大力发展以生态旅游为重点的第三产业。加大旅游资源和要素整合力度，培育服务龙头集团，打响统一品牌。抓好汤旺河国家公园、嘉荫恐龙公园、五营森林公园等景区建设，加快乌马河西岭、达沃斯小镇、梅花山、回龙湾、兴安湖等旅游度假区和房车与自驾车营地的规划与建设，全力打造全省一流、全国知名的精品景区、精品度假区。加快推进五星级宾馆及快捷宾馆、家庭旅馆建设，提升旅游接待能力。继续“提升夏季、突破冬季、拓展春秋”，创新旅游业态，深入开发养生、避暑旅游产品，积极开发森林高尔夫、温泉洗浴、自驾游、房车游等新业态高端旅游产品，推动旅游业升级。加快开发冬季产品，做大滑雪、狩猎、冰壶、雾凇、冰洞等冬季旅游产品的影响力和竞争力。积极培育和发展金融、物流、信息、会展、创意等现代服务业，强化对第二产业的配套服务功能。加强科技创新和服务体系建设，科技成果转化率力争达到75%以上。努力破解融资瓶颈。全面加快融资体系建设，加强银政企之间的交流合作，整合资源，做大市级融资平台。建立健全市和县区两级担保和资源共享的信用体系，鼓励引导各林业局及优质企业出资设立小额贷款公司。认真做好农信社的改制重组工作，争取1-2家商业银行和股份制银行在我市建立分支机构。积极推进中小企业互助联保贷款的实施，扩大银行信贷业务保证范围。支持兴安红酒业上市。推进森林、林木和林地使用权的合理流转，争取早日实现林业资产证券化。

（二）强化生态保护和资源利用，在巩固扩大生态建设成果上实现新突破

抓住国家启动实施《大小兴安岭林区生态保护与经济转型规划》和天保二期工程的重大机遇，加大生态保护建设力度，促进低碳、绿色发展。全面停止森林主伐。国家决定从今年开始，在可采资源基本枯竭的大小兴安岭林区停止森林主伐。这是党和国家着眼于维护生态安全、应对气候变化和加强我国木材资源战略储备做出的重大抉择，也是我们多年的愿望。作为新中国开发最早的林区，一改60多年的生产方式，告别主伐，进入了森林经营时代，这将是中国林业史上的重要里程碑。一方面,我们必须自觉、主动、坚决地把主伐停下来，努力用实际行动偿还对大山的欠债，为国家的生态建设作出新贡献，为子孙后代留下绿水青山，为伊春的可持续发展培好根基。这是我们毫不动摇的政治态度。另一方面,我们必须正视和解决好停伐带来的不利影响，用最短的时间消除停伐带来的阵痛。通过发展林下经济，开发公益性岗位解决林业职工就业岗位不足问题；通过扩大对外采伐，整合林产工业解决原料不足问题；通过争取和运用好大小兴安岭生态功能区建设及天保二期政策解决经费不足问题。进一步强化资源培育和保护。坚持严管资源，确保林木不损失，林地不流失。继续加强对森林、湿地、野生动物及蓝莓、药材等林下资源的保护，维护生物多样性。积极调整树种结构，抓好森林抚育补贴试点，推进标准化苗圃建设，今年完成造林绿化22.01万亩。强化森林防火能力建设，提高林业有害生物科学防治水平，确保森林资源安全。整体推进伊春林区森林认证工作，不断提高森林经营水平。坚持依法用地、节约集约用地，开展对城镇存量建设用地的清理，探索建立土地单位面积产出的评价机制，推动土地资源保护与保障发展和谐共进。加快实施林场所撤并。结合国家政策，大力实施生态移民，统筹解决好林场所撤并后居民的就业、增收以及中心林场、小城镇的规划建设问题。今年，力争全面完成规划的55个林场所的撤并任务，并启动新一轮撤并计划。深入抓好节能减排。大力发展循环经济、低碳经济，积极培育环保型、清洁型产业，加快松花江流域水污染防治规划项目实施进度，抓好钢铁、电力、冶金、建材等重点行业节能降耗，建立重点监控企业污染物排放台账。推广工业、建筑、交通和公用事业节能，积极引进节能、节水和综合利用技术改造项目。加强再生资源回收利用。强力推进烧柴改革，抓好20个试点林场生物质炭、气化及朗乡10万吨生物质成型燃料等项目建设，同步加快生物质发电、生物质柴油等生物能源产业项目开发进程，建立低消耗高产出的生物能源开发利用模式。积极争取森林生态价值补偿。巩固和充分利用好森林与湿地资源价值评估成果，争取国家早日把伊春林区纳入生态效益补偿试点，列为碳权交易试点，使我市的林业资源优势尽快转化为经济优势，不断放大生态效应。

（三）深化改革开放，在增强经济发展动力和活力上实现新突破

改革开放是有效化解体制机制矛盾，推动经济社会发展，加快发展方式转变的重要支撑，我们必须不断深化改革，扩大开放。深入推进林权改革。全面推进集体林权改革，进一步完善国有林权改革配套政策，鼓励和引导承包户大力发展林下经济，实现林地效益最大化，争取实现扩面。继续深化国企改革。分离企业办社会职能，加快林业局主辅分离和辅业改制步伐。推进光明集团家具公司股改和资产重组。推动国有林场改革。统筹推进重点领域改革。全面推进医药卫生体制改革，整合医疗资源，着力优化医院结构布局、资源配置，加强基层和公共医疗卫生服务体系建设，努力满足群众就医看病需求。继续整合普教和职业教育资源，提高政府办学投入水平；加大对上争取力度，做大伊春职业学院和南岔技师学院，推动普教、幼教和职业教育协调均衡稳步发展。加快县(市)区政府机构改革和政府综合执法改革，建立市公共资源交易平台，推进管办分离。推行机关事业单位进人公开招考制度，认真执行“区编市管”规定，严把进人关，严禁混岗用人、违规进人，确保编制数与财政供养人数相一致。加快发展非公有制经济。落实支持非公有制企业和职工自营经济发展的政策措施，引导民间资本更多地投向优势产业、公共事业和基础设施等领域，促进职工自营经济大发展，实现停伐后林业职工的顺利转岗。大力推进招商引资。围绕重点产业和项目，严格落实各级各部门领导的招商引资责任，确保招商目标兑现。创新招商方式、机制和路径，发挥商会、驻外机构、外来投资者及企业的作用，积极开展更有针对性、更有实效的招商活动，确保全年招商引资到位资金达到 200亿元。积极扩大对外贸易。尽可能搭建平台，引导推动更多的企业走向国际市场。重点推进对俄经贸合作，加快嘉荫口岸过货通道和在俄帕什科沃木材工业园区建设，在俄比罗比詹市建设伊春产品展销平台，进一步扩大和深化与俄方在木材采伐与精深加工、农业、矿产开发和边境旅游等方面的合作，实现沿边开放转型升级。

（四）加强城乡建设，在推进城乡一体化发展上实现新突破

以“四城联创”、建设“三优”文明城市为目标，以“二度城市化”为抓手，推动伊春城乡大变化。认真搞好城市规划建设。着眼于未来，高标准搞好城市总体规划及色彩、道路等专项规划的修编，推进中心城“四区连片”建设，实施“东拓、西连、南进、北开”，以回龙湾大街至兴安湖水库、林都大街、城南高速公路“三条线”为框架，以五星级酒店、达沃斯小镇、西岭生态旅游度假区、梅花河度假区、名人旅游度假区为支撑，以南北山、梅花河、溪水、兴安湖等景区景点为背景、尽快形成生态好、景色美、布局合理、人口相对集中的生态园林型的组团城市基础框架。进一步加快基础设施建设。启动新区路网建设，有序推进老城区街道改造升级工程；启动水上公园引水工程、达沃斯小镇以及一批特色小区建设；启动林都大街绿化景观工程，完成北山公园改造和红松广场、文化公园建设。继续实施“三供两治”工程，加快伊春区生活垃圾焚烧及友好、红星、乌伊岭等地垃圾污水处理工程建设进度。加快交通建设，重点抓好伊绥高速公路建设，确保年内竣工通车；开工建设嘉荫口岸延伸公路，完成前嫩公路伊春段路基工程及路面基层总量30%以上。建设农村公路300公里。争取开工建设汤嘉铁路和南岔至浩良河、铁力至金山屯公路桃山至朗乡段公路。加快推进电网改造，开工建设220千伏伊春变电扩建和110千伏乌伊岭输变电、翠宏山送电线路工程，实现西岭、大西林输变电等农网改造升级。切实抓好小城镇建设。多方筹资、融资，加快完善城镇基础设施，着力打造以汤旺河为首的富有林区特色、宜居宜业宜游的一批生态旅游名镇。扎实推进新农村建设。调整分散的村屯布局，大力加强中心村建设，搞好规划设计和功能分区，加快住房、道路、供水、排水等基础设施建设，推广沼气、生物质燃气等新型能源，提高绿化水平，保持村容整洁，进一步改善农村人居环境。与此同时，坚持建管结合，不断巩固扩大国家卫生城创建成果，整体提升城乡品位。

（五）竭力为民办实事，在推进社会事业发展和民生改善上实现新突破

大力发展社会事业，保障和改善民生是维护社会和谐稳定的重要基石。我们必须高度重视社会事业建设，努力扩大基本公共服务。大力抓好安全生产，坚决遏制重特大事故发生。进一步加强社会治安综合治理，积极启动“六五”普法，营造安定祥和的社会环境。今年，要继续抓好去年已经启动但还需续办的实事的推进落实，同时，再重点办好8个方面28件实事。

一是努力提高职工群众就业和增收水平。（1）加大投入和服务力度，开发公益性岗位1000个，开展各项创业培训1000人，提供小额担保贷款1000万元。（2）多渠道开发就业岗位，实现就业再就业4万人。深入开展“大学生创业行动”，全年高校毕业生就业率达到85%。（3）深入实施“一帮一”扶贫创业工程，困难职工扶贫创业率达到80%。（4）提高政企合一区（局）党委、人大、政府、政协和其他公共岗位工作人员、林业职工工资性收入。

二是切实强化社会保障。（5）筹资4900万元，补足提标后最低生活保障和补助资金缺口，保证按时足额发放。（6）提高城镇基本医疗保险待遇水平，在现有基础上提高2倍，解决好群众因病返贫问题。（7）利用国家医疗专项救助资金，资助城镇低保对象参加城镇居民基本医疗保险，实现低保人群参保全覆盖。（8）落实国家政策，提高企业离退休人员养老金标准，人均月增加140元。对符合条件的老年人按时发放高龄津贴。（9）巩固铁力市农村养老保险试点，将嘉荫县纳入第二批新型农村养老保险试点，逐步解决农村居民老有所养问题。（10）适时提高失业金发放标准。（11）采取政府投入、招商引资、社会捐助等方式，力争建设3家大型社会福利院，提高老年人集中供养能力。（12）投入使用市儿童福利院，让孤残儿童进一步感受到党和政府的关爱。

三是优先发展教育事业。（13）加快普及学前三年教育，启动尚处于空白状态的15个县（市）区（局）的公办幼儿园建设，确保到2013年每个县（市）区（局）都有1所公办幼儿园。（14）积极实施省标准化学校建设规划，年内新增标准化学校16所。（15）市和县（市）区（局）筹资1000万元更新教育教学设备。（16）多方筹资，加大贫困生资助力度，全年资助普通高中和中等职业学校贫困生1000人，资助考入二表以上本科院校的贫困大学生500人。

四是加快发展医疗卫生事业。（17）扩大基本药物实施范围，到年底，公办基层医疗卫生机构全部推行国家基本药物制度。（18）加强社区卫生服务中心建设，全面提高服务功能，力争小病不出社区。

五是繁荣发展文体事业。（19）继续推进森林博物馆、文化馆、科技馆、图书馆等公共文化服务设施建设，新建“农家书屋”40个，乡镇综合文化站4个。（20）继续实施农村电影放映工程，开展好“大森林之声”、“城市之光”文化惠民工程等系列文化活动，扩大公益文化服务的规模和范围，将精神文明建设引向深入。（21）加快标准化体育场馆建设，完成主体工程；筹备建设游泳馆、速滑馆、冰壶馆。

六是着力改善人居条件。（22）加大棚户区改造力度，投资36亿元，新建和维修改造住房300万平方米，新建廉租房8000套，改造农村泥草房2272户、16.2万平方米。（23）投资1200万元，完成市中心城区57栋旧楼的平改坡。（24）启动旧小区改造工程，加快解决旧小区基础设施残缺不全、物业弃管问题。（25）投资12.7亿元，分别提高供水、供热、供气普及率6个、5个和2个百分点，达到73%、67%和77%。

七是加快标准化社区建设。（26）新建2个1000平方米的综合社区服务中心，20个300-500平方米的社区服务中心。（27）在中心城4个区建成社区信息化服务平台，完善社区服务体系。

八是突出抓好“稳价保供”。（28）落实中央和省有关政策，降低流通成本，兑现各种价格补贴。依法加强物价和食品质量监管，确保物价稳定、食品安全。

各位代表，面对新的形势和任务，市政府将牢记使命，认真履行人民赋予的权力，建设好学习型、创新型、实干型、亲民型、和谐型、廉洁型政府，努力提高执行力，努力转变工作作风，努力改善发展环境，勤勉尽责，永不懈怠。深入推进“百企万民评服务”和政府效能建设等活动，落实好行政问责制、政务公开制、服务承诺制、廉政责任制、行政领导对群众信访办事接待制等政府工作制度，自觉接受市委领导，主动向人大报告工作、向政协通报情况，及时办理人大代表议案、批评、意见和政协委员提案，加强与民主党派、工商联、无党派人士和群团组织的沟通。切实做好信访工作，畅通民意来源和民众诉求渠道。强化廉政建设，教育引导各级政府工作人员牢固树立干净干事的廉洁观念。进一步完善权力运行监管机制，重拳整治损害群众利益的突出问题。强化对重点领域和关键环节的监督，严肃查处机关工作人员违纪违法案件，大力营造风清气正的政务环境。

各位代表、同志们，历史的成绩鼓舞着我们，宏伟的事业召唤着我们，人民的期待鞭策着我们。让我们在市委的领导下，凝心聚力，奋发图强，锐意进取，扎实工作，为全面完成今年的各项工作任务，谱写我市“十二五”发展的新篇章而努力奋斗!
